# RETRACTION: A Systemic Review and a Meta‐Analysis on the Influences of Closed Incisions in Orthopaedic Trauma Surgery by Negative Pressure Wound Treatment Compared With Conventional Dressings

**DOI:** 10.1111/iwj.70375

**Published:** 2025-03-21

## Abstract

**RETRACTION:** ZhangD. and HeL. , “A Systemic Review and a Meta‐Analysis on the Influences of Closed Incisions in Orthopaedic Trauma Surgery by Negative Pressure Wound Treatment Compared with Conventional Dressings,” International Wound Journal 20, no. 1 (2023): 46–54, 10.1111/iwj.13835.35535660 PMC9797922

The above article, published online on 10 May 2022, in Wiley Online Library (http://onlinelibrary.wiley.com/), has been retracted by agreement between the journal Editor in Chief, Professor Keith Harding; and John Wiley & Sons Ltd. Following an investigation by the publisher, all parties have concluded that this article was accepted solely on the basis of a compromised peer review process. The editors have therefore decided to retract the article. The authors did not respond to our notice regarding the retraction.

**RETRACTION:** D. Zhang and L. He , “A Systemic Review and a Meta‐Analysis on the Influences of Closed Incisions in Orthopaedic Trauma Surgery by Negative Pressure Wound Treatment Compared with Conventional Dressings,” International Wound Journal 20, no. 1 (2023): 46–54, 10.1111/iwj.13835.35535660 PMC9797922

The above article, published online on 10 May 2022, in Wiley Online Library (http://onlinelibrary.wiley.com/), has been retracted by agreement between the journal Editor in Chief, Professor Keith Harding; and John Wiley & Sons Ltd. Following an investigation by the publisher, all parties have concluded that this article was accepted solely on the basis of a compromised peer review process. The editors have therefore decided to retract the article. The authors did not respond to our notice regarding the retraction.

